# Targeted Secondary Screening for Congenital Hypothyroidism in High-Risk Neonates: A 9 Year Review in a Large California Health Care System

**DOI:** 10.3390/ijns7040081

**Published:** 2021-12-01

**Authors:** Alan B. Cortez, Bryan Lin, Joshua A. May

**Affiliations:** 1 Southern California Permanente Medical Group, Department of Pediatrics, Pediatric Endocrinology, Tustin, CA 92780, USA; alan.b.cortez@kp.org; 2 Kaiser Permanente Department of Research, Pasadena, CA 91188, USA; bryanlin9245@gmail.com; 3 Southern California Permanente Medical Group, Department of Pediatrics, Pediatric Endocrinology, Los Angeles, CA 90027, USA

**Keywords:** neonatal screening, congenital hypothyroidism, thyrotropin, infant, newborn, infant, very low birth weight, heart defect, congenital, Down syndrome

## Abstract

Secondary screening for missed congenital hypothyroidism (CH) has been introduced sporadically, but its necessity and optimal strategy have not been recognized. We hypothesized that a simple clinical protocol (performed by a medical group without a governmental mandate) targeting infants at high risk for missed CH can identify cases. We performed a 9-year retrospective review of 338,478 neonates within a California health plan following the introduction of thyrotropin (TSH) secondary screening for neonates at high risk for missed CH due to very-low-birthweight (VLBW), hospitalized congenital heart disease (CHD), and same-sex multiples (SSM). Screening performance by day 60 of life was 95% successful for VLBW and >50% for CHD and SSM, leading to an additional 35% CH treated cases despite re-testing only 1.7% of the cohort. Infants with VLBW or CHD were 33 times more likely (190 times more likely for CHD with Down Syndrome) to receive treatment for CH than random infants diagnosed by primary screening (*p* < 0.001), and 92% of these infants were not found by primary newborn screening. Currently, permanent disease has been documented in 84% of CH by primary screening compared to 27% by secondary screening (*p* < 0.001). This targeted secondary screening program identifies and treats additional CH cases after TSH-only newborn screening.

## 1. Introduction

Congenital hypothyroidism (CH) treatment shortly after birth effectively prevents intellectual disability [1,2,3]. Most screening strategies use a postnatal thyrotropin (TSH) cutoff (on all infants or a subset with low thyroxine levels) to report a presumptive positive result [2]. The following situations, however, may escape diagnosis due to a delay or lack of TSH elevation:

1. Infants with prematurity/low birthweight [4,5,6,7,8,9,10,11,12], cardiac anomalies [5,13,14], or Down syndrome (DS) [15,16,17].
2. Monozygotic twins (same-sex twinning has been an adequate surrogate marker for screening) due to antenatal sharing of blood [18,19].
3. Central hypothyroidism [20,21] through inappropriately low TSH.

The first category does not have a clear causation although hypothalamic–pituitary immaturity and exposure to corticosteroids or dopamine (substances that lower TSH) have been proposed [22,23]. Conversely, exposure to iodine for procedures [3,24] and recovery from sick euthyroid syndrome [25,26] may transiently increase TSH in infants without CH. Various screening strategies to capture missed CH cases have been reported differing in timing (2–12 weeks of life), frequency (1–3 screens), TSH cutoff (>6 to >20 mIU/L), and use of thyroxine results [1,5,8,9,12,13,27,28,29]. 

The last two categories have well-understood physiologic causes [18]. Clinical recognition of central hypothyroidism will occur in many cases due to association with signs of hypopituitarism such as craniofacial/midline defects, genetic diagnoses, recurrent hypoglycemia, recalcitrant hypotension, or micropenis. Screening programs that report low thyroxine levels in absence of TSH elevation might also detect central hypothyroidism [1]. 

Most previous studies have investigated national or regional databases for infants with very low birthweight or congenital heart disease and have suggested a prevalence of CH at least 10 times the rate described for the general infant population [12]. Despite the documented risk for these and other groups, most infants do not receive mandated universal or targeted secondary screening [2,12,30,31]. We now report on targeted secondary screening in the long-term clinical experience of a large group practice which sought to identify more infants at risk for intellectual disability due to CH. 

## 2. Materials and Methods

The clinical protocol started in 2009 at Kaiser Permanente Southern California facilities, a health care system serving >4.6 million people with approximately 37,000 annual births at 14 plan and 4 contracted hospitals. The physicians were members of the Southern California Permanente Medical Group that provides exclusive care to patients residing in Southern California enrolled in Kaiser Health Plan. Human subjects approval was obtained through the Kaiser Permanente Southern California Institutional Review Board. Initial and ongoing education was provided to all pediatricians and neonatologists to implement the secondary screening protocol. 

The California Newborn Screen (“primary screen”) for hypothyroidism consists of TSH testing >12 h of life with abnormal cutoff >29 mIU/L. The targeted TSH secondary screen was developed by the Southern California Permanente Medical Group to clinically manage their patients and was not associated with the California Newborn Screening Program or specific guidelines from a professional society. The screening was performed at approximately 2 weeks of life at the SCPMG Regional Reference Laboratory using the Abbott Architect TSH assay on spun serum for same-sex multiple gestation (SSM) infants, infants with congenital heart disease (CHD) admitted to the Neonatal Intensive Care Unit (NICU), and very low birthweight infants ≤ 1500 g (VLBW). VLBW infants also received TSH screenings at 4 and 6 weeks. Infants with TSH values ≥10 mIU/L on secondary screens were considered abnormal (CH cases) if confirmed on follow-up testing. A pediatric endocrinologist was consulted on every positive case prior to instituting treatment. The clinical protocol relied on the relative simplicity of using TSH measurements due to:

1. The familiarity of pediatricians and neonatologists with the primary screen in California based solely on TSH.
2. The high prevalence of hypothyroxinemia without CH for the low-birth-weight population in the NICU.
3. The current practice of the 15 pediatric endocrinologists in the medical group that relies almost entirely on abnormal TSH and not thyroxine or thyroid imaging to make a diagnosis of presumed primary hypothyroidism.

The rationale of using VLBW rather than prematurity, a TSH specifically ≥10 mIU/L rather than other cutoffs, and the specific times for all the screenings were based on interpretation of the available data at the time [2,3,4,5,6,14,15,16,18]. Thyroid imaging and urinary iodine testing were rarely clinically performed, so are not part of the analysis.

We searched electronic medical record data between 2010 and 2018 for all births and TSH measurements in high-risk infants in the first 60 days of life. International Classification of Diseases-9 and -10 diagnosis codes identified CH, all forms of congenital heart disease (excluding persistent remnants of fetal circulation), multiple gestation, and DS. We also sought evidence of active Kaiser insurance (and hospital admission for CHD and VLBW) at screening times (“available for secondary screening”). For VLBW, the first screen was expected at approximately day 14 of life (days 7–21), the second screen around day of life 28 (days 22–35) and the third screen around day of life 42 (days 36–60). The data collection for the third screen was extended because borderline results at 6 weeks potentially required an additional test. After finding every infant started on levothyroxine in the first 60 days of life, manual chart reviews confirmed this finding and divided these infants into four groupings:

1. CH by the California primary screen
2. CH by the secondary screen per the clinical guidelines
3. Infants started on levothyroxine who were screened or diagnosed due to incorrect or unanticipated use of the secondary screening guidelines
4. Infants diagnosed with CH (often central) due to clinical suspicion (not by primary or secondary screens).

Infants were further categorized as to their demographic and laboratory information, combinations of high-risk categories, which screening in VLBW infants led to levothyroxine treatment, and presence of DS (a known risk factor for CH) [15,16]. We determined whether CH was transient or permanent when sufficient longitudinal data were available. Permanence was defined as TSH ≥ 10 mIU/L after 12 months (whether still on levothyroxine treatment or after discontinuation at 12–36 months leading to TSH ≥ 10 mIU/L) or continued levothyroxine after four years [2].

Continuous characteristics were summarized with median (IQR) and categorical characteristics were calculated as frequencies/percentages. The chi square test for independence was used to compare the difference in CH prevalence, rates for primary and secondary screening, rates by risk factors and DS diagnosis, frequency of screening, degrees of TSH elevation and permanence of hypothyroidism. Relative risks and 95% confidence intervals were calculated to evaluate differences in CH prevalence for primary and secondary screening rates by risk factors and DS diagnosis. Statistical tests were performed at α = 0.05 level, using two-sided tests.

## 3. Results

**Figure 1:**
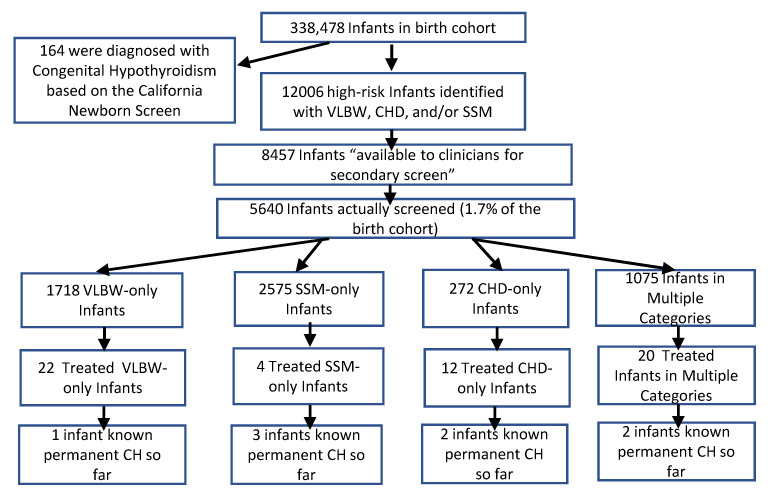
Overview of data collection and outcomes.

The overall flow of the data collection and numbers of infants found in the main categories described in more detail below are outlined in Figure 1.

### 3.1. Secondary Screening Performance

The 338,478 births investigated are described in Table 1. The reported race/ethnicity in this cohort differs from California census data with more Hispanic and Other and less White and Asian (*p* < 0.001, data not shown) [32]. Of 12,006 infants in the high-risk screening categories, 8457 (70%) were available for secondary screening (Table 2) with 1117 (13%) in two or three of the high-risk categories. Completion of screening differed by high-risk category with physicians performing most successfully in the VLBW category with 95% success rate (76% receiving all three screens and 19% receiving one to two screenings, data not shown). Screening CHD and SSM infants was more challenging, at 58.2% and 66.0% respectively. We also found that a large percentage of CHD were screened by the providers because they likely recognized their VLBW status rather than their CHD status (40.8% screened with CHD-only (272 out of 666) vs. 98.1% for CHD with VLBW (514 out of 524). In total, 19% of patients screened were in two or three of the high-risk categories despite the combination groups representing only 13% of the patients available for screening, suggesting better success screening infants with multiple risk factors (*p* < 0.001).

### 3.2. Screening Results for Congenital Hypothyroidism

The prevalence of CH found by the primary screen (Table 3) was 1:2064 infants, congruent with published data in California and the United States [32,33,34]. In contrast, the prevalence of CH discovered by the secondary screen was 1:47 for CHD infants and 1:66 for VLBW infants (increasing to 1:35 for VLBW ≤ 1000 g). CHD-only had the highest CH risk at 1:23 infants. Complete or partial atrioventricular septal defects accounted for 86% of CHD cases with CH by the secondary screen. Only 8% of high-risk infants were identified with CH by primary screening. The effect of a second risk factor (Table 4) greatly increased the CH risk for SSM infants, likely given a higher prevalence of other stronger risk factors (CHD or VLBW) and did not affect the risk for VLBW or CHD infants, already strong risk factors. CHD-only patients had a higher prevalence of CH than those with multiple risk factors, but the data does not suggest to us that VLBW or SSM were somehow protective against CH when CHD was present. Risk in this study is based on retrospective analysis rather than prospective analysis where specific associations rather than causations may influence the apparent risk. In this sample, CHD-only was 25% of the CHD cohort leading to a relatively low denominator yet has >50% of the CH cases leading to a high numerator and thereby creating the appearance of higher risk for CHD-only. The underlying reason was likely that over half the cases of CHD were associated with DS (12 out of 21, data not shown), and most of these DS cases (10 out of 12) were CHD-only. 

Demographic characteristics for gender, race/ethnicity, and maternal age were not significantly associated with CH cases (data not shown). Low gestational age was too closely associated with VLBW to be useful for analysis as an independent variable. 

**Figure 2:**
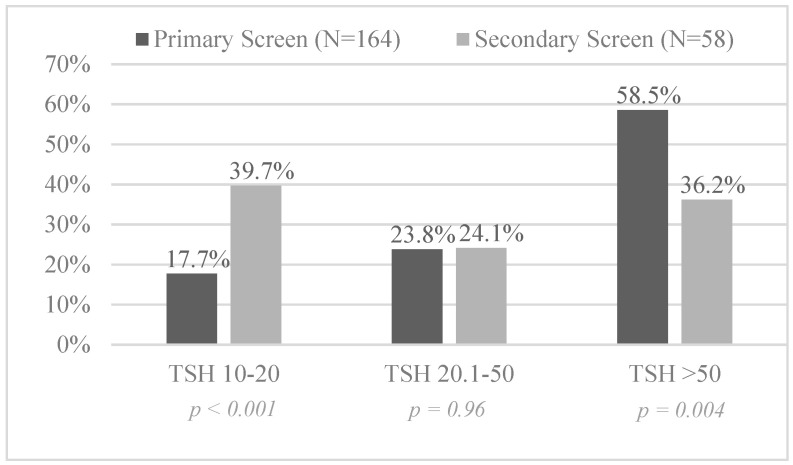
Comparison of initial TSH (mIU/L) levels at time of diagnosis of CH in patients detected on primary vs. secondary screening.

The median day of levothyroxine initiation was day 30 (21, 45) of life with a median TSH of 25.6 (15.4, 86.2) mIU/L for those treated based on secondary screening. Of note, the median TSH on the primary screen for this group was only 4.8 (3.1, 8.9) mIU/L. There were fewer CH cases with TSH >50 mIU/L (36.2% vs. 58.5%) found by secondary screening (Figure 2) compared to primary screening. Timing of levothyroxine treatment in VLBW infants was evenly distributed among the three secondary screens (39.0%, 34.1%,26.8%, respectively, *p* = 0.50) indicating potential utility for each time frame.

### 3.3. Permanence and Transience of Identified CH Infants

**Figure 3:**
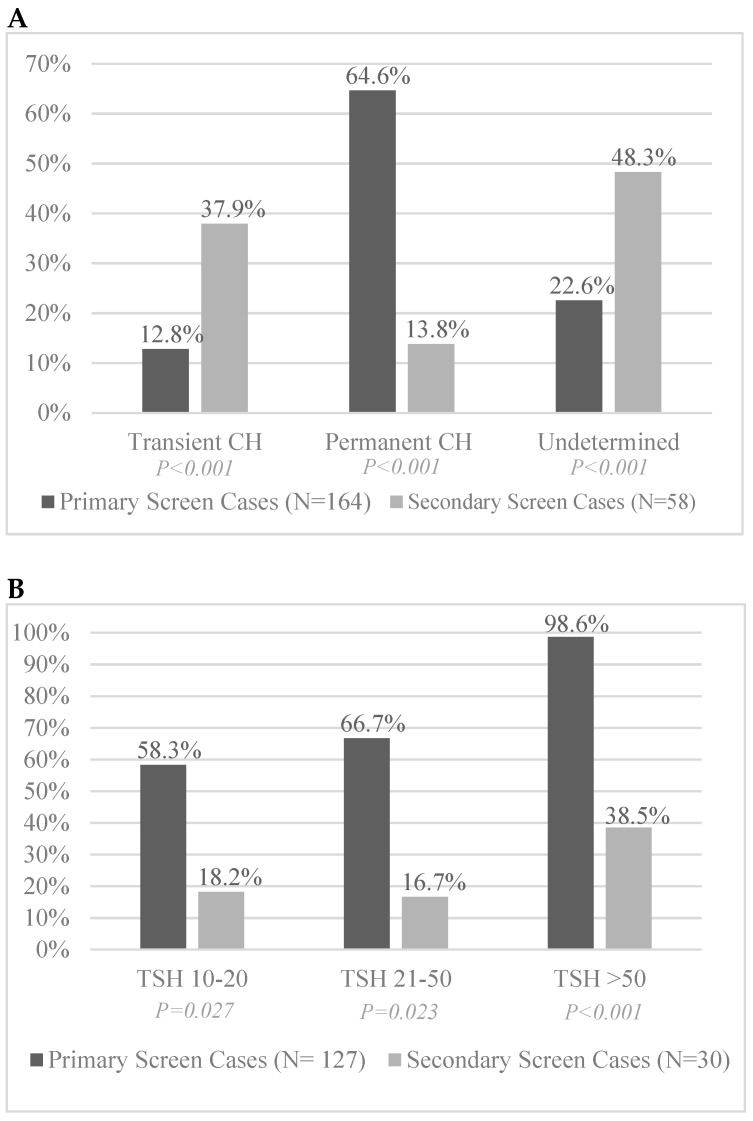
(**A**) Comparison of permanence of CH cases when diagnosed on primary vs. secondary screening. (**B**) Comparison of the likelihood of permanent CH in primary vs. secondary screen positive patients based on degree of TSH (mIU/L) elevation at time of diagnosis. Note: Includes only those patients where adequate data was able to establish potential permanence.

Permanence of CH could not be established for all treated infants due to age, loss of insurance, lack of records, or death. CH by primary screening had an 84% chance of permanent hypothyroidism (127 CH cases met criteria for evaluation), while those with CH on secondary screening had a 27% chance of permanent hypothyroidism (30 CH cases met criteria to evaluate for permanence). Due to patients lost to follow-up or death, 16 of 58 CH cases found on secondary screen could not be evaluated for permanence and 12 of 58 CH cases found on secondary screen were unable to be evaluated due to age < 4 years at the end of the study period (Figure 3A). At least 28% of the CH cases will never have final data due to death (10 of 58) or lack of available records (6 of 58). A secondary screen TSH result >50 mIU/L (at any of the three secondary screens) led to only a 39% chance of permanent hypothyroidism compared to a 99% chance on the primary screen (Figure 3B).

### 3.4. Levothyroxine Treatment Outside of the Screening Protocol

We also found screening and diagnosis of hypothyroidism outside the established protocols (Table 5). Seven of 11 infants treated for suspected hypopituitarism based on symptoms were found to have permanent central hypothyroidism. Application of the secondary screen outside recommended guidelines led to 30 infants starting on levothyroxine, although three of them (all with DS) have confirmed permanent CH. An additional 10 infants were briefly started on levothyroxine for other clinical reasons.

### 3.5. CH with Down Syndrome in the Screened Population

While 26% of infants with DS qualified for secondary screening, seven other DS infants had secondary screening outside the protocol that led to CH treatment. Infants with DS who also had CHD, VLBW, or SSM had an extremely high risk for CH at 1:12 (8.3% vs. 0.08% for non-DS infants, *p* < 0.001). Their rate of permanent CH (8 of 10 could be evaluated) was similar to their non-DS counterparts (114 out of 179, *p* = 0.30). Compared to the general risk of CH on the primary screen for those without DS (1:2098), the risk of CH for CHD infants with DS (12 out of 132) was 190 times greater (*p* < 0.001). Assuming the DS infants who did not receive secondary screening (approximately 50% of the total with DS) did not have CH, the likelihood a DS infant would be diagnosed with CH in the first 60 days of life was at least 75 times greater than our general cohort (3.6% vs. 0.07%, *p* < 0.0001), and the vast majority (88%) were not diagnosed on primary screening.

## 4. Discussion

This multicenter, retrospective review of a large cohort of demographically diverse infants assigned to a single medical group and health plan provides important insight into the virtue of adopting secondary screening programs for CH, specifically infants who receive a TSH-only newborn screen. Most previous studies observed CH cases in these high-risk groups identified by mandated programs, and many of these authors recommended wide acceptance of secondary screening [4,5,6,7,8,9,10,11,12,13,14,15,16,17,18,19]. It is not possible to strictly compare studies because of differences in race/ethnicity which affect CH prevalence, different NICU practices that affect TSH levels, iodine sufficiency in a particular region, and variation in protocol for both the primary and secondary screens. Of note, the main study that did not observe missed CH cases [35] differed from this protocol in looking at only VLBW with one repeat sample at 6 weeks and a TSH cutoff of ≥15 mIU/L. They also did not find differences in developmental outcomes in those with transient elevations compared to matched controls, but their assessments may not have detected subtle changes. 

We found VLBW and CHD infants are at high risk of being missed on the primary screen and, despite sub-perfect screening performance, are much more likely to be diagnosed with CH on the secondary screen compared to the primary screen. We did not investigate reasons for the lower screening performance in the SSM and CHD groups but speculate it would improve with better recognition of monochorionic twins (smaller cohort than same-sex) and that milder forms of CHD are a risk factor. We also found missed SSM cases have higher CH prevalence than that expected for singletons presumably due to the higher risk of concurrent VLBW or CHD (76% of SSM cases) and the fact that twins in general share common environmental and genetic factors (especially monozygotic twins) [19,36]. Two of the SSM-only cases we identified were in a single twin pairing (not consistent with blood admixture as the cause, suggesting such an environmental or genetic connection and potentially explaining the 3.2-fold higher risk seen in SSM-only infants. 

Higher incidence of CH in TSH-only primary screen states, such as California, has been observed compared to those states with a thyroxine/TSH primary screen, though states which couple the thyroxine/TSH primary screen with a universal secondary screen may capture more CH cases [30]. Our study describes a TSH-only primary screening strategy coupled with a targeted TSH-only secondary screening strategy resulting in the identification of an additional 35% of CH cases, with 27% of those additional cases permanent so far. These results suggest that a targeted secondary screening strategy for <3% of the birth cohort may overcome a shortcoming of TSH-only primary screening- missing CH cases in infants with delayed TSH rise. The strategy (without the use of thyroxine values) was not designed to detect central hypothyroidism cases but clinical suspicion was able to identify cases within our cohort consistent with published expectations of 1:20,000 to 1:50,000 [20]. We did not address more complex thyroxine/TSH screening strategies to detect rarer genetic forms of central hypothyroidism. 

With secondary screening, questions persist regarding the likelihood of permanent CH, predictive value of the TSH result, use of thyroxine results, role of other patient characteristics to clarify high-risk groups better, and ideal time frames for secondary screening. Thyroxine levels were performed on any TSH result > 6 mIU/L by reflex testing through the clinical laboratory, but we found no evidence in manual chart reviews these levels influenced decision-making with TSH > 10 mIU/L screens. We believe our analysis demonstrates that the frequency of positive results justifies continuing the current screening program, though it does not inform us if different high-risk criteria or a different laboratory strategy would be more useful. DS, birth weight ≤1000 g, maternal age > 35 years (data not shown), and extreme prematurity (data not shown) were associated with a higher frequency of these delayed CH cases, but our data do not directly inform us about incorporating this information into a more focused screening strategy. More education to limit misapplication of the screening protocol would likely prevent unnecessary levothyroxine starts (30 infants).

Our data showed, not unexpectedly, that infants can be in multiple high-risk categories. More risk factors increased the frequency of screening SSM and CHD infants. We obtained more accurate information about each category (e.g., CHD) by looking at the infants with only a single risk factor (e.g., CHD only), and comparing that to combinations of risk factors (e.g., CHD/VLBW, CHD/SSM, or CHD/VLBW/SSM). For example, SSM cases showed higher CH prevalence than SSM-only primarily due to its association with VLBW. CH prevalence in CHD combinations was lower compared to CHD-only likely due to CHD-only being low volume but representing a higher percentage of DS, with resultant increased risk of CH from DS as described in Results. VLBW-only prevalence was similar to any VLBW combination likely due to the high prevalence of VLBW in the cohort and the high prevalence of VLBW in the other two risk groups. The subcategories within the VLBW group were also informative, with extremely low birthweight infants (≤1000 g) having a much higher incidence of CH than the larger VLBW infants (1001–1500 g). Among CHD cases, only 2 of 21 had complex cyanotic defects suggesting the need to look broadly at all CHD for screening and not its most severe forms. 

In VLBW infants, the relatively even distribution by screening time (2, 4, or 6 weeks) of abnormal TSH levels leading to treatment suggests virtue in multiple secondary screens. This aligns with previous findings suggesting that varied protocols of repeat screening from 1–12 weeks [12] all have potential to identify VLBW infants with negative primary screens. Perhaps the varied pathophysiology of VLBW infants with respect to gestational age, associated comorbidities, exposures, and clinical course all lead to variable delays in TSH rise so multiple secondary screens for this select population are needed. With an urgency to identify CH cases as soon as possible, we advocate not waiting more than 2 weeks for the first test given we identified ~1/3 of the cases at 2 weeks. Even children with mild TSH elevations should be treated because the degree of elevation was not predictive of permanence. 

Still, most cases detected on the secondary screen represent transient CH or in some cases “non-hypothyroidism.” Exposures to iodine for procedures associated with birth or NICU treatments may elevate TSH [24,25]. High TSH seen in the recovery phase of sick euthyroid syndrome following acute illness may be another factor to consider [3,26]. Regardless, we cannot anticipate which cases will be transient, how long the hypothyroid state will continue, and what effect the hypothyroid state can permanently have on neurodevelopment [34,37]. Therefore, the relatively high rate of transient CH in our opinion is not a valid argument for deferring secondary screening or delaying treatment when CH is detected. 

The clinical protocol did not seek DS for secondary screening, yet one quarter of the DS population met the criteria for testing and half were tested. We observed a notably high prevalence of CH in DS (8.3%) on the secondary screen beyond the known prevalence on primary screens [15,38]. Without testing all DS infants, we could not determine the relative contribution of DS, CHD, or endocardial cushion defect (with 8 CH cases in DS with CHD). Currently, national screening guidelines in DS recommend evaluation for acquired hypothyroidism starting at 6 months of age [39] and recognize somewhat higher TSH in younger DS infants [17]. Our findings, in conjunction with Purdy et al. [16] suggest screening much sooner due to delayed rise in TSH may have superior value, and van Trotsensburg, et al. [40] show that even with mild TSH elevations, treatment may preserve intellectual development to the extent possible. 

### Limitations

Our analysis is limited by its retrospective design and by clinical work performed by hundreds of providers in many hospitals and offices over a wide geographic area. While there were over 300,000 infants studied, the population treated with levothyroxine in the first 60 days was relatively small (248) limiting our power to interpret results. For example, we lacked the power to perform sub-analyses on some specific individual or combined demographic and clinical variables that may have been of interest (e.g., determining if maternal age or gestational age were independent risk factors or just associated with the high-risk groups). For CHD, we limited our analysis to infants in the NICU with CHD (matching the clinical protocol), so we cannot comment on the risk for all infants with CHD. We also cannot determine if gestational age could have been a superior criterion compared to birth weight but we do believe that with advances in EMR and prenatal care, monochorionic status would be superior criteria to the same-sex designation used here. Lastly, we can only speculate that the population of high-risk infants who were not screened would yield similar results to those screened. 

## 5. Conclusions

Our data support establishing targeted newborn rescreening programs for infants at high-risk for delayed TSH rise in areas of the United States and other countries not currently performing them. This study adds to the body of knowledge that long-term cases of permanent hypothyroidism are found and that targeted rescreening can be performed in clinical practice even without a governmental mandate supporting it. Overall, we found that a clinical program starting at 2 weeks of life in NICUs and pediatric offices found an additional 35% CH cases among 1.7% infants undergoing secondary screening for possible delayed TSH rise in CHD, VLBW, and SSM infants. CHD and VLBW <1000 g emerged as the highest-risk subgroups. So far, 27% of these cases are permanently hypothyroid, confirming these infant groups are useful targets for secondary screening. We also found an extraordinary number of CH cases with Down syndrome, suggesting routine secondary screening of infants with Down syndrome at 2 weeks may be an appropriate recommendation to consider. 

## Figures and Tables

**Table 1:** Demographic characteristics of cohort 2010–2018.

Description	Total Cohort (%)	High Risk Cohort (%)
**Births**	338,478	12,006 *
**Female**	165,186 (48.8%)	5827 (48.5%)
**Race/Ethnicity**		
Non-Hispanic White	88,683 (26.2%)	3044 (25.3%)
Hispanic	142,646 (42.1%)	5593 (46.6%)
Non-Hispanic Black	23,752 (7.0%)	1328 (11.4%)
Asian	38,736 (11.4%)	1566 (13.0%)
Others	44,661 (13.2%)	475 (4.0%)
**Gestational Age**		
Gestational Age < 28 Weeks	2104 (0.6%)	2116 (17.6)
Gestational Age 28–31 Weeks	2849 (0.8%)	2090 (17.4%)
Gestational Age 32–36 Weeks	26,945 (8.0%)	4684 (39.0%)
Gestational Age 37+ Weeks	306,492 (90.6%)	3116 (25.9%)
**Maternal Age**		
Maternal Age ≤ 21 years	27,105 (8.0%)	697 (5.8%)
Maternal Age 22–34 years	235,097 (69.5%)	7578 (63.1%)
Maternal Age ≥ 35 years	76,276 (22.5%)	3731 (31.1%)
NICU ** Admission	34,407 (10.2%)	7273 (60.6%)
**High Risk Category** ***		
VLBW	4541 (1.3%)	4541 (37.8%)
SSM	7520 (2.2%)	7520 (62.6%)
CHD	1496 (0.4%)	1496 (12.5%)
**Down Syndrome (DS)**	704 (0.2%)	183 (1.5%)

* High risk represents 3.6% of the total cohort. ** Neonatal Intensive Care Unit (NICU), Birthweight ≤ 1500 gm (VLBW), Same-Sex. Multiples (SSM), Congenital Heart Disease in NICU (CHD). *** Due to combinations of high-risk categories, the percentage of the high-risk cohort is >100%.

**Table 2:** Secondary Screen Performance.

Categories of High-Risk Infants	Secondary Screening Criteria Met (N)	Available for Secondary Screening (N)	Number of Infants Screened (N)	Available Infants Screened (%)
**All High-Risk Infants** *	12,006	8457	5640	66.7%
**Very Low Birth Weight (VLBW)** **	4541	2844	2698	94.9%
Birth weight: 1001–1500 g	2355	1767	1643	93.0%
Birth weight: ≤1000 g	2186	1077	1055	98.0%
**Congenital Heart Disease in NICU (CHD)**	1496	1401	987	70.4%
**Same-Sex Multiples (SSM)** ***	7520	5513	3208	58.2%
**Subcategories of High-Risk Infants**				
VLBW + CHD	651	524	514	98.1%
VLBW + SSM	816	588	560	95.2%
CHD + SSM	195	195	189	96.9%
VLBW + CHD + SSM	111	95	94	98.9%
VLBW only	3185	1827	1718	94.0%
CHD only	761	666	272	40.8%
SSM only	6620	4847	2575	53.1%

* 1.7% of total birth cohort (N= 338,478) was screened. The total of all subcategories adds up to 12,339. However, any infant in the VLBW + CHD + SSM group (triple risk) also is counted in each double risk combination so true number of high-risk infants is 12,006. ** Screening performance defined as at least 1 of 3 potential screens. *** Screening percent success comparisons in the three main categories: VLBW vs. CHD was 24.5% (*p* < 0.001); VLBW vs. SSM was 36.7% (*p* < 0.001); and CHD vs. SSM was 12.2% (*p* < 0.001).

**Table 3:** Congenital hypothyroidism (CH) cases with relative risk of CH diagnosis by secondary screen for each high-risk category compared to the risk of CH diagnosis found by primary screening for the total birth cohort.

(N = Total Primary Screened; N = Total Secondary Screened)	CH by Primary Screen	CH by Secondary Screen	CH Prevalence by Secondary Screening vs. Primary Screening in Total Birth Cohort (1:2064)	CH Prevalence by Secondary Screening vs. Primary Screening in Total Birth Cohort (1:2064)
	N (Prevalence)	N (Prevalence)	*p*	Relative Risk (95%CI)
**Total birth cohort (338,478; 5640)**	164 (1:2064)	58 (1:97)	<0.001	21.3 (16.0, 28.2)
**Any high-risk category (12,006; 5640)**	5 (1:2401)	58 (1:97)	<0.001	21.3 (16.0, 28.2)
**High-risk without SSM-only (5386; 3065)** *	3 (1:1795)	54 (1:57)	<0.001	36.2 (27.4, 48.1)
**VLBW (≤1500 g) (4541; 2698)** **	1 (1:4541)	41 (1:66)	<0.001	31.3 (22.3, 44.1)
1001–1500 g (2355; 1643)	0 (n/a)	11 (1:149)	<0.001	13.9 (7.5, 25.4)
≤1000 g (2186; 1055)	1 (1:2186)	30 (1:35)	<0.001	59.0 (40.0, 86.2)
VLBW only (3185; 1718)	1 (1:3185)	22 (1:78)	<0.001	26.5 (17.0, 41.1)
VLBW + CHD (651; 514)	0 (n/a)	8 (1:64)	<0.001	32.3 (15.9, 65.0)
VLBW + SSM (816; 560)	0 (n/a)	12 (1:47)	<0.001	43.9 (24.8, 79.0)
VLBW + CHD + SSM (111; 94)	0 (n/a)	1 (1:94)	<0.001	22.0 (3.1, 155.2)
**CHD** *** **(1496; 987)**	2 (1:748)	21 (1:47)	<0.001	43.9 (28.0, 68.9)
CHD only (761; 272)	2 (1:380)	12 (1:23)	<0.001	89.7 (51.3, 161.6)
CHD + SSM (195; 189)	0 (n/a)	2 (1:95)	<0.001	21.7 (5.5, 87.4)
**SSM (7520; 3208)**	2 (1:3760)	17 (1:189)	<0.001	10.9 (6.6, 18.0)
SSM only (6620; 2575)	2 (1:3310)	4 (1:644)	0.02	3.2 (1.2, 8.6)

* SSM-only excluded to confirm prevalence of CH is higher when delayed rise in TSH (in CHD and VLBW) is the concern for false-negative primary screen rather than blood admixture in multiples. ** The 1 CH cases in triple-risk group is also counted in each double-risk combination, leading to all the categories showing 2 less CH case than the result obtained by adding the subgroups. *** CHD cases included 8 with endocardial cushion defect, 6 with ventricular septal defect, 2 with atrial septal defect, 2 with both atrial and ventricular septal defects, 2 with complex cyanotic disease, and 1 with aortic stenosis.

**Table 4:** Comparison of prevalence of CH diagnosed on secondary screen in those with individual vs. multiple high-risk categories.

Risk Factor(s)	CH Cases/Total Secondary Screen	Difference in Prevalence	*p* *
	Ratio; %; Prevalence	%; (95% CI)	
**VLBW only**	**22/1718 = 1.3% = 1:78**	**Reference**	**Reference**
VLBW/CHD/SSM	1/94 = 1.1% = 1:94	−0.2% (−2.5%, 2.1%)	0.87
VLBW/CHD	8/514 = 1.6% = 1:64	0.3% (−0.9%, 1.5%)	0.61
VLBW/SSM	12/560 = 2.1% = 1:47	0.8% (−0.4%, 2.0%)	0.18
**CHD only**	**12/272 = 4.4% = 1:23**	**Reference**	**Reference**
CHD/VLBW/SSM	1/94 = 1.1% = 1:94	−3.3% (−7.6%, 1.0%)	0.14
CHD/VLBW	8/514 = 1.6% = 1:64	−2.8% (−5.1%, −0.5%)	0.02
CHD/SSM	2/189 = 1.1% = 1:95	−3.3% (−6.5%, −0.1%)	0.04
**SSM only**	**4/2575 = 0.2% = 1:644**	**Reference**	**Reference**
SSM/VLBW/CHD	1/94 = 1.1% = 1:94	0.9% (0.09%, 1.9%)	0.07
SSM/CHD	2/189 = 1.1% = 1:95	0.9% (0.2%, 1.7%)	0.02
SSM/VLBW	12/560 = 2.1% = 1:47	1.9% (1.2%, 2.6%)	<0.001

* All comparisons between any two or three multiple risk categories were not significant- not shown in the table.

**Table 5:** Presumed congenital hypothyroidism cases treated with levothyroxine in the first 60 days of life due to testing or judgement outside of the defined screening process.

Reasons for Treatment	Cases (N)	Known Permanent Cases (N) *
**Secondary screening—unintended use**		
Screening out of guidelines (Down syndrome without risk category)	7	3
Screening out of guidelines (other reasons)	3	0
Diagnosis out of guidelines (TSH 6–10 mIU/L)	11	0
Diagnosis out of guidelines (hypothyroxinemia, normal TSH)	8	0
Diagnosis out of guidelines- other (unaffected twin)	1	0
**Primary screening follow-up issues**		
Treatment for TSH >50 mIU/L but confirmatory test then ruled out CH	4	0
Maternal Graves Disease follow-up	2	0
**Symptomatic screening**		
Hypopituitarism suspected	11	7
Other (neck surgery, hypotonia, hemangioma, genetic syndrome)	4	0
**Total**	51	10

* 5 infants deceased and 11 infants we lack long-term data on levothyroxine use.

## Data Availability

The data involves confidential patient information obtained from their medical records and cannot be shared.

## Abbreviations

CH	Congenital Hypothyroidism
CHD	Hospitalized congenital heart disease
DS	Down syndrome
NICU	Neonatal Intensive Care Unit
SSM	Same-sex multiple
TSH	Thyrotropin
VLBW	Very-low-birthweight
